# Estimating virtual water and land use transfers associated with future food supply: A scalable food balance approach

**DOI:** 10.1016/j.mex.2020.100811

**Published:** 2020-02-24

**Authors:** David O. Yawson

**Affiliations:** Centre for Resource Management and Environmental Studies (CERMES), The University of the West Indies, Cave Hill Campus, Bridgetown, Barbados

**Keywords:** Land scarcity, Water scarcity, Virtual resource, Food security, Climate change, Food balance sheet, Food trade, Barley, United Kingdom

## Abstract

Virtual water or land use is the volume of water or area of land, respectively, used to produce a unit food commodity that is traded. Estimates of future virtual water or land use (as potential mechanisms for mitigating against food insecurity due to resource scarcity) are limited by the need for complex modelling and data requirements regarding trade, for which the data or expertise might be rare or unavailable. This paper presents a simple food balance approach for estimating the status quo food demand and supply and associated virtual water or land use transfers under future conditions. The method is spatially-scalable, accessible to a wider range of users, and illustrated using UK feed barley supply. Key features of the method are:

● Proportionate distribution of a target food item over utilization components is estimated from the FAO Food Balance Sheet of the country of analysis and used to distribute future supply over utilization components.

● The balance between demand and supply is used to estimate the direction and magnitude of virtual water or land use transfers.

● The method can be scaled up from national to regional and global levels and to cover multiple food items.

## Graphical abstract

**Figure:**
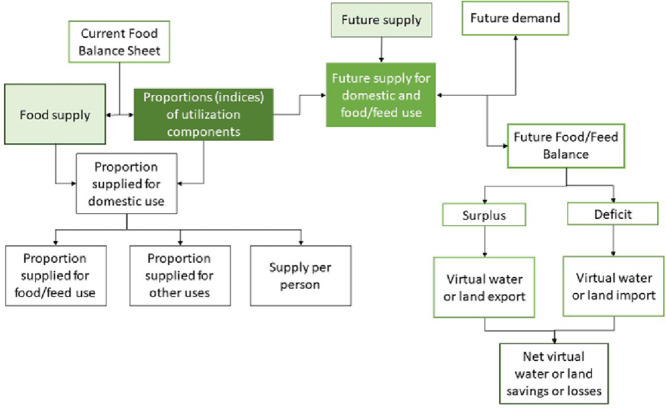

**Table:** Specification Table

Subject Area:	Environmental Science
More specific subject area:	*Virtual water or land use and food security*
Method name:	*Food Balance Approach*
Name and reference of original method:	*Food Balance Sheet (FAO, 2001).*
Resource availability:	*Further information on the method and related data for application can be found at https://discovery.dundee.ac.uk/en/studentTheses/climate-change-and-virtual-water*

## Method details

Prospects of shrinking arable land and water availability, together with rising population, pose formidable challenges to future food security. Food trade will be a crucial component of adaptive responses to future food insecurity [1], [2], [3], [4], especially in land- and water-scarce countries. Food trade is dictated by surpluses and deficits in food production, underpinned by availability of productive resources. The virtual resource concept captures the magnitude of essential productive resources indirectly transferred via food trade. Virtual water [5] or land use [6] is the volume of water or area of land, respectively, used to produce a unit food commodity that is traded. As potential mechanisms for mitigating against food insecurity due to resource scarcity, estimates of future virtual land use and water transfers have value for highlighting the internal and external resource requirements of countries to maintain food security while assessing their environmental impacts. There is a large body of literature on estimates of virtual resource transfers (especially virtual water) under current conditions [7], but very little exist on future estimates. Estimates of future virtual water and land use transfers associated with food supply is constrained by the need for complex modelling for which the data might be unavailable or simply infeasible. In addition, food production and trade are mediated by several factors (political, socio-economic, ecological or environmental) that interact in complex ways across time and space, and thereby complicate reliable estimates of the magnitude of food commodities that will be traded. As a result, there are few estimates of future virtual resource flows (mainly water and land) associated with food commodity trade. There is the need for a simple approach that produces consistent results, which are comparable across varying temporal and spatial scales, to support adaptive policies and decisions. The food balance method presented in this paper addresses this gap as it helps obviate some of these challenges and permits a wide range of stakeholders to make assessments of future virtual land and water transfers associated with food supply.

## Key data required

1. Indices obtained from a current food balance sheet on the target food commodity for the country of interest or analysis. Here, the key information required relates to the proportionate distribution of quantity of food item supplied over utilization components. This information is used to distribute future supply over target utilization components.
2. Future yields of target crop or food item: this could be yields simulated by the analyst or taken from a reliable source. Ideally, this would have taken into consideration projected climate change (or different emission scenarios) especially when crops are considered. Since the crop, simulation model and approach would differ for different analysts, the current report will not provide information on the simulation of the future yield specific to the study reported here. More so, this paper focuses on applying the food balance approach to estimate future food supply and associated virtual land use and water flows.
3. Future land use: with crops, the area of land is multiplied by the yield to obtain total production. The analyst should have access to data on future croplands for the same time period or time slice considered. This could be simulated or obtained from a relevant source. Either this or both (2) and (3) might not be needed if the analyst has the value of total production for the target future time. Where necessary but there are challenges with access to the relevant data, the analyst can use current land area for the target crop as a baseline or business-as-usual scenario, and make scenarios of upward and downward adjustments based on long term mean change in land area. For example, if the long term (at least 10 years) mean change in the area of land for the target crop is 10%, then the analyst can increase and decrease the current land area by 10% at 5% intervals to represent scenarios of future land area. However, this requires an understanding of the factors that drive land use change to justify the adjustments made to represent the future area of land.
4. Population: projected population data can be obtained from National Statistics Authority or from the United Nations’ World Population Prospects data (https://population.un.org/wpp/).
5. Future demand: the analyst should have access to projected future demand per person for the target crop or food item. This is multiplied by the population to obtain total future demand.

## Main steps

1) **Estimate Future Supply and Demand**

The paper proceeds on the assumption that the analyst has the required future production data (or yield and land area if crops are used). In case the analyst has the crop yield and land area for the target future time, then multiply the future yield by the corresponding land area to obtain total production in the future. This represents total domestic production. If multiple scenarios of land use and/or climate change, for example, are used, then the total production for each relevant combination of land use and climate change scenarios should be obtained. Next, multiply the projected population by the projected demand per person to obtain total demand in the future. If multiple scenarios of population and/or climate change are used, for example, then this calculation needs to be done for each combination of scenarios.

2) **Estimate the Food Balance (The Food Balance Approach)**

The FAO Food Balance Sheet (FBS) [8] is a food accounting tool that shows the balance between national food supply and utilization for a given reference year. As a result, the FBS is widely used in the estimation of national food balances (shortages and surpluses) and future food requirements to support policies and strategies regarding food security [8], [9], [10], [11]. The FBS is easily accessible and its wider spatial and temporal coverage permits consistent international comparisons. In the FBS, total supply of a given food item for a given reference year is the sum of domestic production and import, adjusted for changes in stocks. This supply is then accounted for on the utilization side according to quantities exported, used for animal feed and seed, processed for both food and non-food purposes, lost, and finally, the fraction available for human consumption [8]. From here, supply per person is obtained as the ratio between the fraction available for domestic use or human consumption by the population. Further, kcal supply per person is obtained by applying appropriate food composition factors for supplies of all primary and processed products available for human consumption [8].

The food balance approach relies on indices of proportionate distribution of food supply over utilization components, derived from the FBS, which is accessible at http://www.fao.org/faostat/en/#data/FBS. To start with, take the FBS of the target country (country of analysis) for a given reference year from the FAOSTAT database. Select the country of analysis and the aggregated food items of interest from the left panel. From the top right panel (under Elements), select all available options except Protein supply and Fat supply. Finally, select the reference year (preferably the most current year) and proceed to download the data. Open the data in MS Excel and save it as an Excel workbook. Process and structure the data in a way that the elements and their corresponding values are in adjacent columns. Proceed to derive the food balance indices from the FBS as follows:

(1) $$\left(a\right)\;\%\;\text{Domestic}\;\text{Use}\;\left(\text{DU}\right)=\frac{TD}{TP}\times 100$$

Where TD is the total supplied for domestic use (tonnes) and TP is the total domestic production (tonnes).

(2) $$\text{(b)}\;\%\;\text{Food}\;\text{or}\;\text{Feed}\;\text{Use}\left(\%\text{FU}\right):\frac{TF}{TD}\times 100$$

Where TF is the total supplied for food or feed use (depending on whether the analysis is on food or feed).

(3) $$\text{(c)}\;\text{Per}\;\text{capita}\;\text{supply:}\;\text{This}\;\text{will}\;\text{be}\;\text{provided}\;\text{in}\;\text{the}\;\text{FBS;}\;\text{otherwise}\;\text{derive}\;\text{it}\;\text{as}\;\frac{TD}{RP}\times 100$$

Where RP is the total population in the reference year (also provided on the FBS). Note that the method described here stops at food supply and does not extend to dietary energy supply (kcal) even though the method can be applied if this is the interest.

In the food balance approach, it is assumed that the proportionate distribution of the utilization components derived here would remain unchanged to the future time of interest. Be aware that this assumption confers both strength and weakness on the approach. The strength is that we can have an indication of the future food supply situation assuming the current distribution remains the same. This eases the analytical process and makes the latter accessible to a range of interest holders or users. The weakness is that, obviously, many factors (political, economic, environmental, technical) mediate and can alter the proportionate distribution of the sub-components of the total supplied for domestic use. For example, change in economic conditions could in turn change the demand for food, feed or industrial use of a given crop or food item.

Now, apply the indices (values) obtained from Eqs. (1)–(3) to the future total production to obtain the corresponding values of utilization proportions for the future. For example, quantity supplied for domestic use in the future can be estimated as:

(4) $$DU_{f}=\%DU*TP_{f}$$

Where *DU_f_* is the quantity supplied for domestic use in the future, *TP_f_* is the total production value in the future.

For example, if the answer in Eq. (1) is 85%, then calculate *DU_f_* as [(85/100) * TP_f_].

Accordingly, continue to apply the remaining equations to obtain the remaining quantities. If, for example, multiple climate change and/or land use scenarios are being used, then this should be done for each scenario using the respective total production values as a starting point.

Once the proportions of utilization components are calculated, proceed to obtain the future balance between supply and demand as the difference between the total quantity supplied for feed or food use in the future and total demand in the future. A positive answer indicates surplus and a negative answer indicates deficit. Again, this needs to be calculated for all relevant combinations of scenarios if multiple scenarios are used.

3) **Estimate Future Virtual Land Use and/or Virtual Water**

Assuming that surpluses would be exported, and deficits will be balanced by import, the status quo virtual water and land use can be estimated. First, estimate the area of land required to produce the quantity of food equal to the observed deficit (or used to produce the observed surplus) in the country of analysis:

(5) $$DLR\;\left(ha\right)=\frac{D}{Y}$$

Where *DLR* is the domestic land requirement; *D* is the observed deficit (tonnes); *Y* is the yield in the country of analysis (tonnes/ha).

Subsequently, virtual land use can be estimated as:

(6) $$VLU_{i}\left(ha\right)=\sum_{i=1}^{n}\frac{I_{i}}{Y_{i}}$$

(7) $$VLU_{e}\left(ha\right)=\frac{E}{Y}$$

Where *VLU_i_* and *VLUe* are virtual land use import and export, respectively; *I* is the quantity (tonnes) of barley to be imported from country *i* as a share of the observed total deficit in the future); *Y_i_* is the yield of barley (tonnes/ha) in partner country *i; E* is the observed surplus, representing the quantity (tonnes) exported from the country of analysis (note that this is total virtual land export, not disaggregated according to partner countries. If disaggregation is of interest, then *VLU_e_* should be the sum of proportionate export to the number of partner countries considered); *Y* is the yield (tonnes/ha) in the country of analysis.

In case of deficits, then the contribution to land losses or savings could be assessed as the difference between Eqs. (5) and (6). To this end, a positive value would indicate a contribution to global land savings (i.e. land required to produce that quantity domestically is larger than the aggregate land area used to produce the same quantity in the partner countries) and vice versa. In other words, a positive value indicates that the net land productivity (yield) in the country of analysis is higher than the aggregate productivity of the partner countries. Conversely, this can be done for a situation where surplus is observed. Again, this can be done for different combinations of population, climate, and land use scenarios.

To identify trade partner countries to execute the equations above, the following procedure is used.

i. Obtain the main trade partners of the country of analysis with regard to the crop or food item under consideration from the FAOSTAT detailed trade matrix (http://www.fao.org/faostat/en/#data/TM).
ii. From the top-left panel (Reporter Countries), select the country of analysis.
iii. In the bottom-left panel (*Elements*), select ‘Import Quantity’ and ‘Export Quantity’.
iv. In the top-right panel (*Partner Countries*), select all countries (these are the trade partners that import from or export to the country of analysis.
v. In the bottom-right panel (*Items*), select the crop or food item of interest
vi. In the final panel (*Years*), select about three to five years, starting from the most current year backwards, and then proceed to download the data.

Process and structure the data in MS Excel and identify the top partner countries for import and export. These are countries that could account for about 90% or more of the total import or export, respectively. To obtain the proportionate contribution of each partner country to future import to the country of analysis, take the total import from these top partner countries as 100% and, subsequently, estimate the contribution of each partner country (as a percentage of the sum of import from the selected top partner countries). Then, multiply the percentage for each partner country by the total deficit to obtain the proportionate quantity to be imported from each country and this is the value used for *Ii* in Eq. (6). Conversely, the same approach can be applied to obtain the proportion quantity exported to each partner country in case of surplus. To obtain *Y_i_* (yield in the partner countries) and to avoid complicated, time-consuming simulations (though this is recommended where feasible), the following procedure is used:

i. Obtain a 10-year time period (or more) yields for both the country of analysis and the identified partner countries from FAOSTAT production data (choosing yield for the countries of interest and for the crop under consideration).
ii. Empirical relationships (regression equations) between the yields of the country of analysis and each partner country can be developed and used to predict future yields in the partner countries based on the observed future yields in the country of analysis. Where this is unreliable, differences between the yields in the country of analysis and each partner country for each of the 10 years can be calculated. The mean of these differences (positive or negative) is then added to the yield of the country of analysis to obtain the adjusted or estimated yield in the future for the respective partner countries.

A similar approach is used to estimate the status quo virtual water flows.

i. Estimate the virtual water content of the crop or food item in the country of analysis:
  (8) $$VWC\left(m^{3}/\text{ton}\right)=10\left(\frac{ET_{c}}{Y}\right)$$

where *ET_c_* is the crop evapotranspiration; *Y* is the yield in the country of analysis; 10 is a scalar to ensure consistent units.

ii. Based on the same surplus and/or deficit (as above), estimate the status quo virtual water flows:
  (9) $$TVW_{e}=VWC\;\times \;Q_{e}$$
  (10) $$TVW_{i}=\sum_{i=1}^{n}VWC_{p,i}\;\times \;Q_{i}$$

Where *TVW_e_* and *TVW_i_* (m^3^) are total virtual water exported from country of analysis and imported to country of analysis, respectively; *VWC_p,i_* is the virtual water content of the crop or food item in partner country *i; Q_e_* and *Q_i_* are quantities of the crop or food item exported from the country of analysis (same as the surplus) and imported from partner country *i* (which sum up to the deficit), respectively.

The *TVW_e_* is easily estimated since *Q_e_* is the total surplus in the country of analysis and the associated VWC is obtained from the simulated yield. However, for the *TVW_i_*, the procedure explained for virtual land use above can be used to obtain an adjusted VWC of the crop or food item for the respective partner countries, or by making assumptions based on anticipated water productivities in the partner countries. Current VWC values can be obtained from the WaterStat Database of the Water Footprint Network (www.waterfootprint.org) [12].

## Method application

The food balance approach is now demonstrated using future supply of barley for feed use in the United Kingdom (UK) and associated virtual land use in the 2050s.

1) **Future Supply and Demand**

For reasons stated earlier, this paper will not present the details of the future yield simulations (see [10,13] for more details). The data in Table 1 (available, together with sources or estimates, in [10]) will be used to illustrate the application of the food balance approach.

**Table 1:** Parameters and values for estimating future supply and demand. Source: [10].

1) Mean yield (under the medium emission scenario)	7.24 tonnes/ha
2) Land area (under the Mid scenario)	963,000 ha
3) Population (Constant Fertility Scenario)	80.3 million
4) Projected per capita feed barley demand per year	153 kg
From Table 1:
Total future production (*TP_f_*, see Eq. (4)) would be (1) × (2) = 7.24 × 963,000 =  6,972,120 tonnes.
Total future demand would be (3) × (4) = 80.3 million × 153 kg = 12,285,900,000 kg =12,285,900 tonnes.

2) **Indices of Food Balance**

Table 2 shows the calculation steps for the indices derived from the UK FBS for the reference year 2009.

**Table 2:** Calculation steps for deriving baseline indices from the FBS (Source: [3]).

Item Number	Description	Value
(1)	Total domestic production	5964 thousand tonnes
(2)	Total export	633 thousand tonnes
(3)	Total import	115 thousand tonnes
(4)	Total supplied for domestic use	4953 thousand tonnes
(5)	% domestic use (% DU, Eq. (1))	(5) = [(4)/(1)] × 100 = 83.0%
(6)	Total supplied for animal feed	3037 thousand tonnes
(7)	% feed use (% FU, Eq. (2))	(7) = [(6)/(4)] × 100 = 61.3%
(8)	Total supplied for brewing and distilling (considered collectively as ‘malt use’)	1713 thousand tonnes
(9)	% malt use (Eq. (2))	(9) = [(8)/(4)] × 100 = 34.6%
(10)	Self sufficiency	(10) = [(1)/(4)] × 100 = 120.4%
(11)	Per cap barley supply	(11) = [(4)/total population*] = 80 kg year^−1^
(12)	Per cap feed barley supply	(12) = [(6)/total population] = 49 kg year^−1^
Table 3 shows the proportionate quantities of utilization components in the future (applying the obtained percentages to TP_f_).

**Table 3:** Estimates of future quantities of utilization components.

Item Number	Description	Value
(1)	Total domestic production (TP_f_)	6,972,120 tonnes
(2)	Total supplied for domestic use in the future (DU_f_)	83% of TP_f_ = 5,786,859.6 tonnes
(3)	Total supplied for animal feed in the future (FU_f_)	61.3% of DU_f_ = 3,547,344.9 tonnes
(4)	Total supplied for brewing and distilling (‘malt use’)	34.6 of DU_f_ = 2,002,253.4 tonnes
(5)	Barley supply per cap	0.072065 tonnes or 72 kg
(6)	Feed barley supply per cap	0.044176 tonnes or 44 kg
The difference between feed-use supply and demand = 3547,344.9 − 12,285,900 = −8,738,555.1 tonnes (deficit).

## Future virtual land use

Now that the balance between supply and demand has been obtained, the future land area required to produce the quantity of barley equal to the observed deficit is estimated using Eq. (5): $DLR\;\left(ha\right)=\frac{D}{Y}$ = 8,738,555.1/7.24 = **1,206,982.7**

Next, Eq. (6) is used to estimate virtual land use import (*VLU_i_*) to balance the deficit: $VLU_{i}\left(ha\right)=\sum_{i=1}^{n}\frac{I_{i}}{Y_{i}}$

To estimate *I_i_* (yield in a partner country), the FAOSTAT trade matrix data showed that eight countries accounted for about 95% of total UK barley import (see Table 4). Taking the total import from these eight partner countries as 100%, the percentage share of each country to this total was estimated. The percentage shares were then multiplied by the observed deficit to obtain the proportionate quantity of barley that will be imported from each partner country in the future. To obtain the yields for each partner country, the observed average national yield for each of the top eight partner countries and the UK, for the period 1998 – 2017 was retrieved from the FAOSTAT database (under production, crops: http://www.fao.org/faostat/en/#data/QC). Because regression equations were unreliable, the differences between the yield of each partner country and the UK for each was calculated. The mean (positive or negative) of the differences for each partner country was added to the future UK yield to obtain the adjusted or estimated yield for the respective partner country. Finally, the area of land required in each partner country to produce the estimated share of barley import to the UK was then calculated using Eq. (6).

**Table 4:** share of barley import from partner countries.

Partner country	(1) Baseline share of barley import to UK (%)	(2) Estimated future yield (tonnes/ha)	(3) Share of future import to the UK (tonnes)	(4) Virtual land use (ha)
Ireland	47	8.4	(1) * total observed deficit; = (47/100) * 8738,555.1 = **4,107,120.9**	= (3)/(2) = 4107,120.9/8.4 = 488,943.0
France	17	7.7	1,485,554.4	192,929.1
Germany	13	7.6	1,136,012.2	149,475.3
Ukraine	7	3.8	611,698.9	160,973.4
Spain	5	4.2	436,927.8	104,030.4
Denmark	4	6.8	349,542.2	51,403.3
Sweden	4	5.8	349,542.2	60,265.9
Italy	3	5.1	262,156.7	51,403.3
**SUM**				**1,259,423.7**

Now, the net virtual land use (NVLU, ha) can be estimated as Eqs. (5) and (6) using the answers or values obtained for these equations:

$$DLR\;\left(ha\right)=\frac{D}{Y}=1,206,982.7$$

*Total VLU_i_* (ha) = 1,259,423.7

Therefore, NVLU (ha) = 1,206,982.7 – 1,259,423.7 = **−52,441**

This is interpreted as a contribution to global land loss as the total land required to produce the quantity of the deficit is smaller than the total virtual land use import.
